# Psychometric Properties and Clinical Usefulness of the Youth Self-Report DSM-Oriented Scales: A Field Study among Detained Male Adolescents

**DOI:** 10.3390/ijerph13090932

**Published:** 2016-09-21

**Authors:** Olivier F. Colins

**Affiliations:** Department of Child and Adolescent Psychiatry, Curium-Leiden University Medical Center, Endegeesterstraatweg 27, Leiden AK 2342, The Netherlands; o.colins@curium.nl; Tel.: +31-71-515-9615

**Keywords:** mental health, screening, antisocial, offenders, detained, psychiatric, forensic, ASEBA

## Abstract

It is unknown if the DSM-oriented (DSM) scales of the Youth Self-Report (YSR) are useful to determine what kind of narrowly-focused psychiatric assessment is needed, and how well these scales serve as a triage tool in real-world forensic settings. To address this knowledge gap, the YSR and diagnostic interviews were administered to 405 detained boys as part of a clinical protocol. Continuous DSM scale scores (e.g., Conduct Problems) were moderately to highly accurate in predicting their corresponding disorder (e.g., conduct disorder), whereas dichotomized DSM scale scores were not. To test the DSM scales’ usefulness for triage purposes, the sensitivity and specificity of being in the borderline range of one or more DSM scales were calculated. Almost all boys who did not have a disorder were in the normal range of at least one DSM scale (high specificity). However, many boys with a disorder would have been missed if such a decision rule was used for triage purposes (low sensitivity). In conclusion, their relations with the corresponding disorders support the construct validity of the DSM scales in an applied forensic setting. Nevertheless, the findings also warrant against the use of these scales for planning further narrowly-focused assessment or for triage purposes.

## 1. Introduction

Notwithstanding that the prevalence of psychiatric disorders among detained boys is high (e.g., [1]), juvenile detention facilities all too often lack budgets, time, and/or qualified personnel to perform in-depth psychiatric assessments. Juvenile detention staff, therefore, may have a special interest in mental health screening tools that can provide concrete directions on how to plan further narrowly-focused psychiatric assessments (e.g., only ascertain the presence of major depression disorder if the youth is high on a scale that screens for depressive feelings). Most screening tools, however, are not designed to map psychiatric disorders as defined by classification systems such as the DSM, and include scales that cover many symptoms, moods, and thoughts that are not specific to any particular disorder. As such, it may come as no surprise that the accuracy of these scales to identify detained youth with a specific disorder is most often moderate at best (e.g., [2]). An exception may be the DSM-oriented (DSM) scales of the Youth Self-Report (YSR; [3]). The YSR was designed to be useful in many contexts, including juvenile justice settings [4], and its DSM scales comprise items identified as being very consistent with specific DSM-IV disorders [4]. Even though these DSM scales were not intended to be a perfect equivalent of the disorder(s) under consideration, they still may provide concrete directions for further assessment.

### 1.1. Empirical Studies on the YSR DSM Scales

Very few studies tested the relations between the DSM scales and their corresponding psychiatric disorder(s). The DSM Affective Problems scale seems to be a highly accurate screener for major depression disorder (MDD) in clinic-referred youth [5,6], but not among general population samples [7,8,9,10]. Among detained youth, the accuracy of this scale as a screener for MDD ranged from poor [11] to moderate [12], making it impossible to draw any conclusions. Findings with regard to the DSM Anxiety Problems scale were far more mixed, with some studies showing that this scale was a moderately to highly accurate screener for anxiety disorder in detained ([12]; but see: [11]) and clinic-referred adolescents ([13]; but see: [6]), though not in clinic-referred children [13]. The DSM Attention Deficit/Hyperactivity and Oppositional Defiant Problems scales were found to be an accurate screener for attention deficit/hyperactivity disorder and for oppositional defiant disorder, respectively [12,13]. The DSM Conduct Problems scale, finally, accurately screened for a conduct disorder diagnosis in detained youth [12]. 

### 1.2. Contribution of This Study

The present study will substantially contribute to the literature on the DSM scales in at least four ways. First, the previous section clearly showed that studies on the screening accuracy of DSM scales other than the Affective and Anxiety Problems scales are quite rare. The present study will fill this void.

Second, studies on the DSM scales typically provided confidentiality of the information and anonymity to its participants. Consequently, it is unsure to what extent results from these studies have field utility and, thus, are informative for clinicians who are working with detained youth. When being asked to complete the YSR as part of a clinical protocol, detained youth, indeed, may be reluctant to reveal information (e.g., tendency to act out aggressively) that is unknown to others and can be used against them (e.g., in court). To address this issue, the present study relied on data that were gathered during a clinical protocol, thus, outside of a research context.

Third, a large number of detained youth score high on various scales of a mental health screening tool, and meet diagnostic criteria for two or more psychiatric disorders [14]. Consequently, testing relations between a DSM scale and its corresponding disorder might be more relevant from a psychometric perspective (i.e., construct validity) than from a clinical perspective. Indeed, it can be argued that may be the best a mental health screening tools can offer when working with detained youth is to do an initial sorting or triage of detained youth into one group that includes almost all of the youth with psychiatric disorders (so they can further be assessed) and one group that has as few youth as possible with psychiatric disorders (and that need no further assessment) [15]. Therefore, this study also examined how the DSM scales serve as a triage tool.

Fourth, studies on the DSM scales did not test if results differed across youth from various ethnic origins. This is unfortunate (a) because detained ethnic minority youth are overrepresented in detention facilities, and often report less mental health problems than detained ethnic majority youth (e.g., [16]); and (b) because the screening accuracy of mental health screening tools can vary across ethnic groups (e.g., [14,17,18]). To fill this void, the current study differentiated between youth from various ethnic origins.

## 2. Methods

### 2.1. Setting and Participants

Data stem from male adolescents from two large youth detention centers in The Netherlands. These data were gathered as part of the standardized mental health screening and comprehensive assessment provided to each boy entering these institutions (for details see: [2]). For the purpose of this study, data for 443 boys who completed the YSR between July 2009 and August 2011 were made available to the author (All detained boys who entered the two facilities in this time span were asked to complete the YSR, which implies that YSR administration did not depend on certain inclusion criteria such as presenting symptoms or results from previous tests). For eight boys, information about ethnicity was missing, and 30 boys did not complete the diagnostic interviews described in Section 2.2. Therefore, all analyses relied on data from 405 boys. The mean age of this sample (*n* = 405) was 16.69 years (SD = 1.40; ranging 13 to 24 years, with only 5% being 19 years or older). With regard to ethnicity, 23.7% were from Dutch origin, 27.4% from Moroccan origin, 22.0% from Surinamese or Antillean origin, and 26.9% from other origins (e.g., Turkish).

### 2.2. Measures

**The Youth Self-Report.** The Dutch YSR [19] consists of 118 items referring to behavioral and emotional problems during the past six months. These items must be answered on a 3-point scale ranging from 0 (not at all) to 2 (often). Since 2001, the YSR has six DSM scales that are based on the international experts’ judgments about the extent to which YSR items correspond with DSM symptoms. Specifically, the Affective Problems scale (13 items; e.g., “I cry a lot”) covers symptoms of major depressive disorder and dysthymia; the Anxiety Problems scale (6 items; e.g., “I worry a lot”) symptoms of generalized anxiety disorder, separation anxiety disorder, and specific phobias; the Somatic Problems scale (7 items; e.g., “Aches or pains”) symptoms of somatic disorders; the Attention Deficit/Hyperactivity Problems scale (7 items; e.g., “I fail to finish things that I start”) symptoms of attention deficit/hyper-activity disorder; the Oppositional Defiant Problems scale (5 items; e.g., “I argue a lot”) symptoms of oppositional defiant disorder; and the Conduct Problem scale (15 items; e.g., “I am mean to others”) symptoms of conduct disorder. In the present study raw, continuous DSM scale scores were used unless otherwise specified.

**DSM-IV disorders.** The Diagnostic-Interview Schedule for Children-Fourth Version (DISC-IV) is a psychiatric diagnostic interview that covers many DSM-IV psychiatric diagnoses, can be administered by trained non-clinicians [20], and was used to assess the past year’s prevalence of attention deficit/hyperactivity disorder, oppositional defiant disorder, conduct disorder, alcohol use disorder, marijuana use disorder, and substance use disorder other than alcohol or marijuana use disorders. The Development and Well-Being Assessment (DAWBA) is a psychiatric diagnostic interview that is administered by trained non-clinician interviewers [21]. The DAWBA was used to assess major depressive disorder, posttraumatic stress disorder, panic disorder, agoraphobia, and generalized anxiety disorder. For all these disorders, the DAWBA uses a past four-week time-frame, except for generalized anxiety disorder (past six months). Finally, based on all the aforementioned DISC and DAWBA generated diagnoses, an additional variable was created (labelled “Any Psychiatric Disorder”) to identify boys who met criteria for at least one disorder. Of note, DISC and DAWBA generated diagnoses were based on information that was gathered during the DISC and DAWBA interviews, which implies that the diagnoses were not contaminated by knowledge of or information collected by means of the YSR scales.

**Ethnic background.** Using the Dutch standard classification of ethnic groups (Dutch Central Bureau of Statistics), boys were categorized as “Moroccan” or “Antillean or Surinamese” when the boy and/or at least one parent were born in Morocco or Dutch Antilles or Surinam, respectively. If both parents were of non-Dutch origin, the mother’s country of birth determined the child’s ethnicity. Boys were classified as Dutch when both parents and the child were born in the Netherlands. All other boys were assigned to the Mixed ethnicity group.

### 2.3. Procedure

Graduate students and test assistants with a Master’s degree trained by clinical researchers administered the YSR to the youths and performed the comprehensive assessments (most often on the same day the YSR was completed). Assistance was available at request (e.g., if the youth did not understand a question). When reading abilities were insufficient, the questionnaires were read to the youth. Youths were aware that this was part of the youth detention centers’ routine and that the outcomes are available to detention staff. Through standardized information provided by the youth detention centers upon start of detention, youths and their parents/caretakers were informed that these outcomes would be used—unless they refused—for scientific research. Given that routine screening and assessment was part of clinical care, the relevant boards of the youth detention centers waived the requirement to obtain informed consent from youth and—for youth younger than 18—parent(s)/caretaker(s). A university Institutional Review Board deemed study protocols to be exempt from review because we used de-identified data that was collected as part of a clinical protocol, and for clinical purposes. 

### 2.4. Data-Analytical Strategy

First, mean scores (SD) for the DSM scales, and the percentage of boys in the borderline and clinical range [3] were calculated. Group-wise comparisons (with a Bonferonni correction) were performed next to test for significant differences in mean scores and percentages between the four ethnic groups. The DSM scales are based on the international experts’ judgments, and not on factor analyses as is the case for the YSR empirical scales. Yet, to ensure that mean scores can be meaningfully compared, measurement invariance (MI) tests are required. Due to sample size issues MI could not be performed. Therefore, the group-wise comparisons should be considered exploratory, and their results should be interpreted with caution.

Second, to test the internal consistency of the DSM scales, three indices were calculated, being Cronbach’s alpha (α), mean inter-item correlation (MIC), and mean corrected- item-to-total correlation (MCITC). Alphas were interpreted as follows: <0.60 = insufficient, 0.60 to 0.69 = marginal, 0.70 to 0.79 = acceptable, 0.80 to 0.89 = good, and 0.90 = excellent [22]. MIC values should be at minimum in the range of 0.15 to 0.50 [23], whereas the MCITC should >0.30 [24] to be considered adequate. 

Third, the relations between the DSM scales and their corresponding disorder(s) were studied in two ways to allow comparison with prior work. Independent one-way ANOVAs were performed to test if boys who meet criteria for a specific psychiatric disorder (e.g., conduct disorder) obtain higher scores on the corresponding DSM scale (i.e., Conduct Problems) than youths without that disorder. Then, the Area under the Curve (AUC) was calculated to test the accuracy of the DSM scales to identify boys with a specific psychiatric disorder, using both continuous and dichotomized (below versus above the borderline cut-off) DSM scale scores. The AUC is based on a combination of sensitivity and 1-specificity, and measures the probability that a DSM scale (e.g., Affective Problems) will yield a higher score for a randomly chosen youth with a disorder (i.e., major depressive disorder) than for a randomly chosen youth without that disorder. An AUC value greater than 0.5 reflects above chance-level accuracy and an AUC value of 1.00 indicates perfect accuracy.). A general guideline is that, for a screening tool to be moderately or highly accurate, AUCs of at least 0.70 or 0.90 are wanted, respectively [25]. 

Fourth, to test if any given (combination of) DSM scales identify boys with any disorder (cf. triage purpose), the sensitivity, specificity, positive predictive value (PPV), and the negative predictive value (NPV) of being in the borderline range on one or two or more DSM scales will be calculated as well. The PPV and NPV were calculated because they may be more appealing for clinicians than the sensitivity and specificity, that is: the PPV(NPV) shows how many detained boys that were (not) in the borderline range of at least one DSM Problem scale will eventually (not) meet criteria for a disorder. All analyses were performed with SPSS 23 (IBM Corp., Armonk, NY, USA) and *p* < 0.05 was used as an indicator of statistical significance.

## 3. Results

### 3.1. Descriptive Information

Mean DSM scale scores are shown in the upper part of Table 1. Group comparisons demonstrated that Dutch boys had significantly higher mean scores on all but one of the DSM scales (i.e., Somatic Problems) than Moroccan boys, and significantly higher scores on the DSM Attention-deficit/Hyperactivity, Oppositional Defiant, and Conduct Problems scales than boys in the Mixed ethnic group. Moroccan boys had significantly lower scores on the DSM Attention-deficit/Hyperactivity, Oppositional Defiant, Conduct, and Somatic Problems scales than Antillean/Surinamese boys. Other between-group differences were: a higher Attention-deficit/Hyperactivity Problem score in Dutch than in Antillean/Surinamese boys; a higher Conduct Problems score in Antillean/Surinamese than in boys in the Mixed ethnicity group; and a higher Affective Problems score in boys from the Mixed group than in Moroccan boys. Table 1 (lower part) also presents the percentage of youths that were in the borderline and the clinical ranges, and shows that there were almost no significant differences between the ethnic groups, with very few exceptions. Prevalence rates for psychiatric disorder diagnoses in the total sample and the four ethnic groups can be found in the Supplementary Material.

### 3.2. Internal Consistency of the YSR DSM Scale Scores

Table 2 shows that the DSM Attention-deficit/Hyperactivity and Conduct Problems scale scores were acceptable or adequately internally consistent according to all three indices. Although αs for the DSM Oppositional Defiant, Affective, Anxiety, and Somatic Problems scales were often insufficient or marginal, the MIC and MCICT values were, overall, in the range to be considered adequate. Of note, the DSM Affective and Somatic Problems scale scores in Dutch boys, the Anxiety Problems scale score in Moroccan boys, and the Somatic Problems scale score in boys from the Mixed ethnicity group were below or just at or above the recommended MIC and/or MCICT values to be considered adequate.

### 3.3. Relations between DSM Scales and Their Corresponding Psychiatric Disorder

Table 3 shows that scores on all DSM scales were significantly higher in the group of boys with (vs. without) the psychiatric disorder of interest. These findings were replicated in each ethnic group, with two exceptions: the Oppositional Defiant Problem score between boys without (No) versus with (Yes) oppositional defiant disorder in the Moroccan and Mixed group (see Table S1 of the Supplementary Material) was not statistically significant. Table 4 shows that when using continuous DSM scale scores, all DSM scales were significant (all *p* < 0.01) and moderately accurate predictors of the corresponding disorder of interest. Overall, this finding was replicated across the four ethnic groups, with one exception: the DSM Affective Problems scale was not significantly related to major depressive disorder in Moroccan boys. The AUCs were not statistically significantly different (*p* < 0.05) across ethnic groups, with one exception, being that the AUC for the DSM Anxiety Problem scale in Moroccan boys (0.91) was significantly higher (*p* = 0.009) than the AUC for this DSM scale in boys from the Mixed ethnicity group (0.66).

The predictive accuracy of all DSM scales decreased when using dichotomized DSM scale scores. Specifically, the AUCs for these dichotomized DSM scale scores were often non-significant and/or generally below 0.70, except for DSM Conduct Problems scale in the total sample, Dutch boys and Moroccan boys, and the Affective Problems scale in Antillean/Surinamese boys. The AUCs were not statistically significantly different (*p* < 0.05) across ethnic groups. As a rule of thumb, there should be at least 10 cases that have the diagnosis, 10 that do not, 10 that test positive (above or at the DSM borderline score), and 10 that test negative (below the DSM scale borderline score) [26]. As can be seen in Tables S2 and S3 of the Supplementary Material, and in contrast to the total sample, these conditions are rarely met in the four ethnic groups. Therefore, the AUC for dichotomized DSM scale scores presented for the four ethnic groups are exploratory and should be interpreted with great caution. 

### 3.4. The DSM Scales as a Triage Tool

Table 5 shows that the likelihood that boys with any disorder were in the borderline range (cf. sensitivity) on at least one DSM scale, ranged from 0.38 (Moroccan boys) to 0.52 (Dutch boys), and on at least two or more DSM scales from 0.12 (Moroccan boys) to 0.27 (Dutch boys). This, for example, means that 46% of the total sample of 196 boys with any disorder was in the borderline range of at least one DSM scale. The likelihood that boys without a disorder were not in the borderline range (cf. specificity) on at least one DSM scale ranged from 0.82 (Mixed boys) to 0.91 (Antillean/Surinamese boys), and on at least two DSM scales from 0.97 (Dutch boys) and 0.98 (all other boys). Thus, boys without a disorder were very unlikely to have a score in the borderline range on one or two DSM scales.

Table 5, finally, presents positive (PPV) and negative predictive values (NPV). For example, a PPV of 0.78 (total sample) means that 78% of the 114 boys who had a score in the borderline range on one or more DSM scales met criteria for at least one disorder. A NPV of 0.77 (Moroccan boys) implies that 77% of the 90 Moroccan boys who did not have a score in the borderline range on one or more DSM scales did not meet criteria for any disorder.

## 4. Discussion

Forensic research has resulted in an increased awareness among clinicians and policy makers of the mental health needs of detained adolescents. Although there are many methods for assessing mental health problems in juvenile justice settings [15], most methods require more time and staff expertise than most youth detention centers can afford. Therefore, brief self-report questionnaires are appealing for youth detention centers, particularly because they can help clinicians to classify incoming youths according to their level of urgency (e.g., [18]). Unfortunately, the overwhelming majority of forensic studies that tested the psychometric properties or ‘clinical’ usefulness of these self-report tools was conducted as part of a research project where the participants were assured that the data would be anonymous and not impact their cases. Subsequently, there is a need for on-going research into the reliability and validity of self-report tools when they are taken out of the lab and into legal settings where the information may bring actual consequences for the informant. The present study filled a gap in the literature by being the first field study that scrutinized the psychometric properties and clinical usefulness of the DSM scales among detained boys.

This study showed that the internal consistency of the DSM scale scores as indicated by α ranged from 0.57 (Anxiety Problems) to 0.81 (Conduct Problems). These αs were identical to the range of αs reported in a previous study among detained boys [12], and in line with a study among clinic-referred adolescents [27]. According to the two other indices (that are less sensitive to the number of items in a scale than α), all six DSM scales scores were at least adequately internally consistent. The finding that the DSM scale scores, overall, are also internally consistent in the three ethnic minority groups bears substantial clinical relevance, as detained youths are most often not from the major ethnicity group of the country where they live and are being detained [28,29]. 

Although caution is warranted (see Section 2.4), Moroccan boys had significantly lower DSM scale scores than boys from Dutch, Antillean/Surinamese, and Mixed ethnicity. This finding dovetails well with recent studies showing that Moroccan detained boys reported lower levels of mental health problems than boys from Dutch and other ethnic origins as measured by various tools, including the YSR [30], the Strengths and Difficulties Questionnaire [16], and the MAYSI-2 [14]. Detained Moroccan boys, thus, seem to systematically report fewer mental health problems than boys from other ethnic origin, regardless of the measure being used. Future studies are warranted to test if different item functioning could explain these cross-ethnic differences in screening scale scores. Importantly, these lower mean scores in detained Moroccan boys do not imply that these youngsters do not have mental health needs that must be addressed. 

This study also showed that the DSM scales are related to their corresponding disorder. The results showed that boys who meet criteria for a specific psychiatric disorder (e.g., conduct disorder) obtain higher scores on the corresponding DSM scale (i.e., Conduct Problems) than boys who were without that disorder. Likewise, the DSM scales were moderately to highly accurate in predicting their corresponding disorder, though not always significantly. These AUCs were somewhat higher than those reported in a prior study in detained boys on the DSM scales [12]. Specifically, in this prior study, the AUC for the DSM Attention Deficit/Hyperactivity Problems scale in predicting attention deficit/hyperactivity disorder in the total sample was 0.77 (vs. 0.85 in this study), for the DSM Oppositional Defiant Problems scale in predicting oppositional defiant disorder 0.78 (vs. 0.81), for the DSM Conduct Problems scale in predicting conduct disorder 0.76 (vs. 0.85), for the DSM Affective Problems scale in predicting Any Affective Disorder 0.65 (vs. 0.78), and for the DSM Anxiety Problems scale in predicting Any Anxiety Disorder 0.73 (vs. 0.76). The accuracy of the DSM scales in real-world settings, thus, are at least as good as those stemming from a study that guaranteed confidentiality to its participants [12]. Overall, these findings suggest that the DSM scales do relatively well in identifying youths with specific psychiatric disorders. 

However, the clinical usefulness of the DSM scales for identifying detained boys who require further narrowly-focused assessments can be questioned, though, for several reasons. First, the AUCs for dichotomized DSM scales in the total sample were generally below 0.70, which is unfortunate because clinicians often find cut-points more appealing and easier to use than thinking in dimensional terms [31]. Second, the high comorbidity rate in detained boys may limit the usefulness in determining what kind of narrowly focussed assessment is needed. Indeed, a boy with comorbid conduct disorder and anxiety disorders may have DSM Conduct and Affective Problems scale scores that fall in the borderline range. Yet, if a clinician would rely on the results being displayed in Table 4, he or she would only refer the boy for further assessment to ensure whether he meets criteria for CD. This would be unfortunate, because comorbidity of externalizing and internalizing disorders increases the risk of poor outcomes, including suicidal behavior [32], and implies that interventions are likely to be insufficient when not adequately tailored to their complex needs [1,33].

The present study, finally, examined if DSM scales can be useful for triage purposes. Although the positive and negative predictive values may be more appealing for clinicians, these values can be affected by prevalence rates of the disorders [34,35], and may therefore difficult to generalize to other samples. The sensitivity and specificity indices reported in Table 5 do not run this danger, and, for example, showed that 98% of the 209 detained boys who were without any disorder were not in the borderline range on at least two DSM scales. This means that only a small number of youths would have been referred for further comprehensive psychiatric evaluation while this was not warranted. Unfortunately, the sensitivity of the multiple decision rules used in the present study was poor, suggesting that a high percentage of the boys with at least one psychiatric disorder would have been missed if these decision rules were used for triage purposes. Of note, a prior study among detained boys showed that an alternative mental health screening tool that was completed during a clinical protocol served relatively better as a triage tool [2]. Clearly, further research is warranted to elucidate how well various mental health screenings can be used for triage purposes. 

The findings of this study must be considered in the context of several limitations. First, the YSR and diagnostic interviews used different time frames, a difference that may partially explain why some DSM scales did not accurately predict the presence of a disorder (see also: [8]). Second, the YSR has no DSM scale on substance use that may help to identify boys with one of the most prevalent disorders among detained adolescents, being substance use disorder (e.g., [1]). Third, the diagnostic interview to assess the externalizing disorders (the DISC-IV) was different from the diagnostic interview to assess internalizing disorders (the DAWBA). Given evidence that diagnoses resulting from these measures are not equivalent [36], future research on the topic should use at least one diagnostic interview to assess all relevant disorders of interest. Fourth, even though parents (and teachers) of detained youths are often difficult to locate or unwilling or unable to provide reliable information [37], the sole reliance on self-report still can be considered to be a limitation of the present study. Fifth, only detained males were included in this study, implicating that this is the only population that an inference can be drawn upon. Sixth, because of sample size considerations, Antillean and Surinamese youths were merged together in one group, whilst youth from various other origins were merged together in the Mixed ethnicity group. Although this approach is in line with previous papers, it may have obscured differences regarding the performance of the DSM scales between these ethnic minority groups. Seventh, due to power issues, the AUCs for dichotomized DSM scale scores presented for each of the four ethnic groups are exploratory at best. Finally, graduate students and test assistants who were present during the YSR administration did not calculate the YSR scores (these were computer generated) and were not necessarily the same ones who interviewed the youth with the DISC or DAWBA. Although this occasionally was the case, it is highly unlikely that they were aware of the youths’ YSR DSM scale score, and even if this was the case this would not have affected the DISC and DAWBA diagnoses for the simple reason that YSR information is not considered in the DISC and DAWBA algorithms. However, an anonymous reviewer argued that the index tests (i.e., YSR DSM scales) and the reference standards (DISC and DAWBA diagnoses) were, strictly speaking, not 100% blinded, and that this must be mentioned as a limitation.

## 5. Conclusions

The present study shows that the DSM scale scores are internally consistent, whilst the positive relations between these scales and their corresponding disorder(s) also support the construct validity of these DSM scales in an applied forensic setting. Nevertheless, the findings also warrant against the use of these scales for planning further narrowly-focused assessment or for triage purposes, at least in Dutch youth detention centers. 

## Figures and Tables

**Table 1:** Mean (SD) DSM scales scores and percentages of youths in the borderline and clinical range.

DSM Scale	Total Sample (*n* = 405)		Dutch (*n* = 96)		Moroccan (*n* = 111)		Antill./Surin. (*n* = 89)		Mixed (*n* = 109)	
	*Mean*	*(SD)*	*Mean*	*(SD)*	*Mean*	*(SD)*	*Mean*	*(SD)*	*Mean*	*(SD)*
ADH	3.51	(2.95)	5.11 ^a,b^*^,c^*	(3.26)	2.43 ^a,e^	(2.60)	3.62 ^b^*^,e^	(2.52)	3.11 ^c^*	(2.75)
OD	1.85	(1.87)	2.41 ^a^*^,c^	(1.96)	1.31 ^a^*^,e^	(1.68)	2.11 ^e^	(1.81)	1.70 ^c^	(1.86)
Conduct	4.03	(3.70)	5.27 ^a^*^,c^	(4.01)	2.79 ^a^*^,e^*	(3.26)	4.88 ^e^*^,g^	(3.72)	3.50 ^c,g^	(3.33)
Affective	2.85	(3.06)	3.61 ^a^*	(2.90)	2.08 ^a^*^,f^	(2.85)	2.88	(3.12)	2.94 ^f^	(3.20)
Anxiety	1.52	(1.68)	1.82 ^a^	(1.85)	1.04 ^a^	(1.35)	1.59	(1.64)	1.70	(1.76)
Somatic	1.27	(1.71)	1.24	(1.57)	0.94 ^e^	(1.53)	1.63 ^e^	(1.93)	1.36	(1.76)
	Bord.	Clin.	Bord.	Clin.	Bord.	Clin.	Bord.	Clin.	Bord.	Clin.
ADH	6.9	2.5	17.7 ^a^*^,b^*^,c^*	6.3	2.7 ^a^*	0.9	3.4 ^b^*	1.1	4.6 ^c^*	1.8
OD	5.2	2.0	8.3	3.1	3.6	1.8	5.6	1.1	3.7	1.8
Conduct	12.1	7.2	18.8 ^a^*	10.4	5.4 ^a^*	5.4	15.7	7.9	10.1	5.5
Affective	7.7	3.0	10.4	2.1	4.5	2.7	7.9	4.5	8.3	2.8
Anxiety	3.0	1.5	6.3	3.1	0.9	0.9	3.4	1.1	1.8	0.9
Somatic	11.4	2.5	12.5	1.0	7.2	0.9	12.4	5.6	13.9	2.8

Notes: Antill./Surin. = Antillean/Surinamese; Bord./Clin. = Borderline/Clinical Range; ADH = Attention/Deficit-Hyperactivity; OD = Oppositional Defiant; Mean scores in cells with similar superscripts refer to statistically significant (*p* < 0.05) differences. Superscripts with an * refer to moderate effect size (*d* ≥ 0.60) and underlined superscripts to strong effect size (*d* ≥ 0.80).

**Table 2:** Internal consistency of the DSM scales.

DSM Scale (#Items) ^a^	Total Sample (*n* = 405)			Dutch (*n* = 96)			Moroccan (*n* = 111)			Antil./Surin. (*n* = 89)			Mixed (*n* = 109)		
	*α*	*MIC*	*MCITC*	*α*	*MIC*	*MCITC*	*α*	*MIC*	*MCITC*	*α*	*MIC*	*MCITC*	*α*	*MIC*	*MCITC*
ADH (#7)	0.79	0.35	0.52	0.80	0.35	0.53	0.79	0.35	0.52	0.70	0.24	0.40	0.77	0.32	0.50
Oppositional Defiant (#5)	0.68	0.30	0.44	0.66	0.29	0.42	0.68	0.31	0.44	0.59	0.22	0.35	0.72	0.35	0.49
Conduct (#15)	0.81	0.25	0.45	0.82	0.26	0.46	0.81	0.26	0.46	0.78	0.22	0.41	0.78	0.22	0.41
Affective (#13) ^b^	0.75	0.20	0.38	0.65	0.14	0.28	0.78	0.25	0.43	0.78	0.26	0.44	0.76	0.22	0.41
Anxiety (#6)	0.57	0.18	0.32	0.64	0.26	0.40	0.47	0.12	0.25	0.55	0.17	0.31	0.54	0.18	0.30
Somatic (#7)	0.64	0.20	0.36	0.53	0.14	0.30	0.66	0.20	0.38	0.68	0.25	0.41	0.63	0.19	0.35

Notes: Antill./Surin. = Antillean/Surinamese; *MIC* = Mean-Intercorrelation Coefficient; *MCITC* = Mean Corrected-Item-To Total Correlation Coefficient; ADH = Attention/Deficit-Hyperactivity; ^a^ Number between parentheses refer to the number of items in the scale; ^b^ Due to zero variance only 12 items were included in this scale to calculate the internal consistency indices in the total sample and the Dutch and Moroccan subgroups (item 77 was excluded), and 11 items in the other two ethnic groups (items 18 and 77 were excluded).

**Table 3:** Construct validity of the DSM scales (Total Sample *n* = 405).

DSM Problems Scale → Disorder	Disorder	
	No	Yes
	Mean ^a^ (SD)	Mean ^a^ (SD)
Attention-deficit/Hyperactivity Problems → ADHD (*n* = 34)	3.16 (2.72)	7.29 (2.70) *
Oppositional Defiant Problems → ODD (*n* = 20)	1.73 (1.78)	4.10 (2.12) *
Conduct Problems → CD (*n* = 72)	3.06 (2.70)	8.51 (4.34) *
Affective Problems → Depression (*n* = 36)	2.50 (2.69)	6.36 (4.20) *
Anxiety Problems → Anxiety Disorder ^b^ (*n* = 65)	1.23 (1.38)	3.01 (2.21) *

^a^ Means are means for the DSM scale presented in the left part of the first column on the same row for youth without or with the disorder presented in the right part of the first column; Number between parentheses are the number of boys with the disorder * *p* < 0.01; all significant differences were strong in magnitude (Cohen’s d between 0.99 to 1.50); ^b^ The YSR DSM Anxiety Problems scale covers symptoms of generalized anxiety disorder, separation anxiety disorder, and specific phobias. Unfortunately, few boys met criteria for generalized anxiety disorder and specific phobia (*n* = 8), while separation anxiety disorder was not assessed because the prevalence can be artificially inflated due to detention itself (i.e., being away from family and friends). In line with prior work on the DSM Anxiety Problems scale, other anxiety disorders—being panic disorder, agoraphobia and posttraumatic stress disorder—were also included in this Any Anxiety Disorder variable (e.g., [11,12,13]).

**Table 4:** DSM scales as disorder-specific predictors (area under the curve with 95% confidence intervals).

Scale → Disorder	Raw (Continuous YSR DSM Scale Scores)					Borderline Range ^a^ (Dichotomized YSR DSM Scale Scores)				
	Total	Dutch	Mor	An/Su	Mix	Total ^b^	Dutch ^b^	Mor ^b^	An/Su ^b^	Mix ^b^
ADH → ADHD	0.85 ***	0.76 **	np	0.86 **	np	0.66 **	0.61	np	10.64	np
	(0.80; 0.91)	(0.66; 0.87)		(0.76; 0.97)		(0.54; 0.77)	(0.45; 0.77)		(0.39; 0.89)	
Opp. Def. → ODD	0.81 ***	0.85 **	np	np	np	0.60	0.70	np	np	np
	(0.72; 0.90)	(0.71; 0.99)				(0.46; 0.75)	(0.48; 0.91)			
Conduct → CD	0.85 ***	0.90 ***	0.92 ***	0.76 **	0.81 ***	0.71 ***	0.78 ***	0.73 **	0.63	0.68 *
	(0.80; 0.91)	(0.81; 0.98)	(0.95; 0.99)	(0.63; 0.90)	(0.70; 0.93)	(0.64; 0.79)	(0.65; 0.90)	(0.55; 0.91)	(0.48; 0.79)	(0.52; 0.84)
Affective → MDD	0.78 ***	0.77 **	0.71	0.90 **	0.71 *	0.67 **	0.66	0.55	0.77 *	0.70 *
	(0.69; 0.86)	(0.63; 0.91)	(0.50; 0.91)	(0.80; 0.99)	(0.49; 0.93)	(0.56; 0.78)	(0.48; 0.84)	(0.32; 0.79)	(0.54; 0.99)	(0.48; 0.91)
Anxiety → Anxiety	0.76 ***	0.73 **	0.91 ***	0.74 **	0.66 *	0.56	0.65 *	0.49	0.56	0.52
	(0.69; 0.82)	(0.60; 0.86)	(0.84; 0.98)	(0.60; 0.89)	(0.52; 0.81)	(0.48; 0.65)	(0.50; 0.80)	(0.31; 0.68)	(0.39; 0.72)	(0.37; 0.67)

Notes: Mor = Moroccan; An/Su = Antillean/Surinamese; Mix = Mixed; ADH = attention-deficit/hyperactivity; np = not presented due to very low numbers of boys with the disorder (see Supplementary Material; * *p* < 0.05; ** *p* < 0.01; *** *p* < 0.001; ^a^ The percentages of boys with scores in the clinical range were too low to perform these analyses whilst using the Clinical Range score (see Table 1); ^b^ the number of boys in the borderline range who were with and without the disorder can be retrieved in the Supplemental Material).

**Table 5:** Sensitivity, specificity, and positive and negative predictive values for being in the borderline range for one or more and two or more DSM scales.

Number of Scales	Any Disorder ^a^			
One or more scales ^b^	Sensitivity	Specificity	PPV	NPV
Total Sample (25 + 89 = 114)	0.46	0.88	0.78	0.63
Dutch (3 + 33 = 36)	0.52	0.90	0.92	0.47
Moroccan (8 + 13 = 21)	0.38	0.90	0.62	0.77
Antillean/Surinamese (4 + 21 = 25)	0.46	0.91	0.84	0.61
Mixed (10 + 22 = 32)	0.41	0.82	0.69	0.59
Two or more scales ^b^				
Total Sample (5 + 40 = 45)	0.20	0.98	0.89	0.56
Dutch (1 + 17 = 18)	0.27	0.97	0.95	0.31
Moroccan (2 + 4 = 6) ^c^	0.12	0.98	0.66	0.71
Antillean/Surinamese (1 + 11 = 12)	0.24	0.98	0.92	0.54
Mixed (1 + 8 = 9) ^c^	0.15	0.98	0.89	0.54

Notes: Numbers between parentheses are: the number of boys in the borderline range without disorders + the number of boys in the borderline range with any disorder = total number of boys in the borderline range; PPV = positive predictive value; NPV = negative predictive value; ^a^ The number of boys with any disorder are: 196 (total sample), 63 (Dutch), 34 (Moroccan), 46 (Antillean/Suriname), and 53 (Mixed; see also Supplementary Material); ^b^ The Somatic Problems scale included; ^c^ The aforementioned rule of thumb [26] suggests that these values must be interpreted cautiously.

## Supplementary Materials

The following are available online at www.mdpi.com/1660-4601/13/9/932/s1, Table S1: Construct validity of the DSM scales in four ethnic groups, Table S2: Prevalence rates of psychiatric disorders, Table S3: Number of boys at or above the borderline range for a DSM scale who were with and without the disorder.
