# Supplementary material for: Psychometric Properties and Clinical Usefulness of the Youth Self-Report DSM-Oriented Scales: A Field Study among Detained Male Adolescents
Source: Int J Environ Res Public Health. 2016 Sep 21;13(9):932. doi: 10.3390/ijerph13090932 (PMC5036764; doi:10.3390/ijerph13090932)
Supplement: Supplementary file 1 [file ijerph-13-00932-s001.pdf]

Supplementary Materials: Psychometric Properties and Clinical Usefulness of the Youth Self-Report DSM-Oriented Scales: A Field Study among Detained Male Adolescents

Olivier F. Colins

Table S1. Construct validity of the DSM scales in four ethnic groups.

DSM Problems Scale → Disorder	Dutch (*n* = 96)		Moroccan (*n* = 111)		An/Sur (*n* = 89)		Mixed (*n* = 109)	
	Disorder		Disorder		Disorder		Disorder	
	No	Yes	No	Yes	No	Yes	No	Yes
	Mean [a] (SD)	Mean [a] (SD)	Mean [a] (SD)	Mean [a] (SD)	Mean [a] (SD)	Mean [a] (SD)	Mean [a] (SD)	Mean [a] (SD)
ADH Problems → ADHD	4.58 (3.17)	7.59 (2.53)	2.24 (2.41)	6.40 (3.43)	3.32 (2.26)	7.14 (2.97)	2.90 (2.58)	7.40 (2.88)
Oppositional Defiant Problems → ODD	2.13 (1.72)	5.11 (2.26)	1.27 (3.50)	3.50 (0.71)	2.02 (1.76)	4.00 (2.16)	1.65 (1.87)	2.60 (1.52)
Conduct Problems → CD	3.75 (2.44)	10.09 (4.25)	1.99 (1.97)	8.85 (4.58)	4.16 (3.24)	7.53 (4.25)	2.81 (2.68)	7.23 (4.04)
Affective Problems → Depression	3.17 (2.51)	6.46 (3.64)	1.91 (2.65)	4.57 (4.50)	2.46 (2.74)	7.71 (3.45)	2.62 (2.73)	6.55 (5.46)
Anxiety Problems → Anxiety Disorder	1.41 (1.34)	3.40 (2.58)	0.84 (1.21)	3.00 (1.15)	1.31 (1.44)	2.87 (1.96)	1.47 (1.48)	2.79 (2.50)

[a] Means are means for the DSM scale presented in the left part of the first column on the same row for youth without or with the disorder presented in the right part of the first column; all differences were statistically significant except for the Oppositional Defiant problem Score between boys without (No) versus with (Yes) ODD in the Moroccan and Mixed group.

Table S2. Prevalence rates of psychiatric disorders.

Psychiatric Disorder	Total Sample (*n* = 405)	Dutch (*n* = 96)	Moroccan (*n* = 111)	An/Sur (*n* = 89)	Mixed (*n* = 109)
	n (%)	*n* (%)	*n* (%)	*n* (%)	*n* (%)
Any Disorder	196 (48.4)	63 (65.6)	34 (30.6)	46 (51.7)	53 (48.6)
Attention-Deficit/Hyperactivity Disorder	34 (8.4)	17 (17.7)	5 (4.5)	7 (7.9)	5 (4.6)
Oppositional Defiant Disorder	20 (4.9)	9 (9.4)	2 (1.8)	4 (4.5)	5 (4.6)
Conduct Disorder	72 (17.8)	23 (24.0)	13 (11.7)	19 (21.3)	17 (15.6)
Depression	36 (8.9)	13 (13.5)	7 (6.3)	7 (7.9)	9 (8.3)
Anxiety Disorder	65 (16.0)	20 (20.8)	10 (9.0)	16 (18.0)	19 (17.4)

Table S3. Number of boys at or above the borderline range for a DSM scale who were with and without the disorder.

DSM Problems Scale → Disorder	Total		Dutch (n = 96)		Moroccan (n = 111)		An/Sur (n = 89)		Mixed (n = 109)	
	Disorder		Disorder		Disorder		Disorder		Disorder	
	Below [a]	Above [a]	Below [a]	Above [a]	Below [a]	Above [a]	Below [a]	Above [a]	Below [a]	Above [a]
ADH Problems → ADHD	16	12	11	6	1	2	1	2	3	2
Oppositional Defiant Problems → ODD	16	5	4	4	4	0	4	1	4	0
Conduct Problems → CD	15	34	4	14	0	6	7	7	4	7
Affective Problems → Depression	17	14	5	5	4	1	3	4	5	4
Anxiety Problems → Anxiety Disorder	3	9	0	6	1	0	1	2	1	1

[a] Numbers are Numbers for the DSM scale presented in the left part of the first column on the same row for youth without or with the disorder presented in the right part of the first column.

© 2016 by the author; licensee MDPI, Basel, Switzerland. This article is an open access article distributed under the terms and conditions of the Creative Commons by Attribution (CC-BY) license (http://creativecommons.org/licenses/by/4.0/).
